# Different Types of Management for Anastomotic Leak Post Esophagectomy

**DOI:** 10.7759/cureus.48404

**Published:** 2023-11-06

**Authors:** Noor S Basendowah

**Affiliations:** 1 Surgery, Faculty of Medicine in Rabigh, King Abdulaziz University, Jeddah, SAU

**Keywords:** sealants, evac, stent, esophagectomy, anastomotic leak

## Abstract

Esophagectomy is a critical surgical procedure for managing various esophageal disorders, including malignancies, strictures, and reflux disease. Nonetheless, it is associated with significant postoperative complications, with anastomotic leak being a major concern. An anastomotic leak is defined as an unintended communication failure between the proximal esophageal segment and the conduit after surgery. This review explores evolving strategies for managing anastomotic leaks after esophagectomy, including factors contributing to leaks, such as patient-related, surgical, and perioperative factors. Diagnostic advancements, encompassing clinical evaluation, radiological imaging, and endoscopy, enable rapid and accurate detection, which is crucial for timely intervention. Management approaches have evolved beyond surgical revisions and now include conservative measures, antibiotics, and endoscopic therapies, particularly for high-risk surgical cases. Novel approaches, such as endoscopic stents, tissue sealants, and regenerative therapies, hold promise in revolutionizing treatment and outcomes. Prevention strategies, encompassing patient optimization, surgical techniques, and perioperative care, are key in minimizing leak occurrence. A multidisciplinary approach involving various specialists is essential for tailored treatments and optimized outcomes. In conclusion, anastomotic leaks post esophagectomy remain a significant challenge, and this review provides a comprehensive resource on evolving management strategies, from conservative measures to innovative techniques, assisting clinicians in decision-making for leak management.

## Introduction and background

Esophagectomy is a complex surgical procedure crucial for treating various esophageal disorders, including malignancies, strictures, and reflux disease. However, it carries postoperative complications, with anastomotic leak being a major concern [1].

Anastomotic leak refers to unintended communication failure between the proximal esophageal segment and the conduit after surgery, potentially leading to severe infections, sepsis, and more. Rates of anastomotic leak after cervical and thoracic anastomosis vary in the literature, with reported rates ranging from 6.6% to 17.2% and 2% to 15.9%, respectively, with associated mortality rates between 7.2% and 35% [2-7]. Despite surgical advances, managing leaks remains crucial.

Factors contributing to leaks, including patient-related and surgical factors, are discussed, emphasizing the importance of understanding their etiology and risk factors to improve preventive strategies [7]. Diagnostic advancements, including imaging and biomarker identification, enable rapid and accurate detection, vital for timely intervention [8]. Management has shifted beyond surgical revisions, encompassing conservative approaches, antibiotics, and endoscopic therapies, particularly for cases with high surgical risks [8]. Novel approaches like endoscopic stents, tissue sealants, and regenerative therapies are emerging, promising to revolutionize treatment and outcomes [9].

Prevention is key, with perioperative strategies and enhanced recovery protocols aimed at minimizing leak occurrence [10]. Additionally, understanding long-term implications on survival, quality of life, and function is crucial. Given the complexity, a multidisciplinary approach involving various specialists is vital. Collaboration among surgeons, gastroenterologists, radiologists, nutritionists, and infectious disease specialists is essential to tailor treatments and optimize outcomes.

## Review

Etiology and risk factors

Anastomotic leaks following esophagectomy are influenced by a complex interplay of patient-related factors, surgical techniques, and perioperative care protocols. A thorough understanding of these etiological factors and risk elements is indispensable for risk stratification, patient counseling, and the implementation of preventive strategies. By addressing these factors proactively, healthcare professionals can contribute to a reduction in the incidence of anastomotic leaks and ultimately enhance patient outcomes after esophagectomy [11].

Patient-Related Factors

Several patient-related factors contribute to the occurrence of anastomotic leaks, including comorbidities like diabetes, cardiovascular diseases, chronic obstructive pulmonary disease, and obesity, which impair wound healing, tissue perfusion, and the immune response. Atherosclerotic calcification of supplying arteries is an emerging risk factor. Malnutrition, especially preoperative malnutrition and low albumin levels (<3.0 g/dL), increases the risk [3]. Patients with a BMI over 30 kg/m² (obesity) or under 18.5 kg/m² (underweight) are also at higher risk [12]. Smoking and excessive alcohol consumption delay wound healing and tissue perfusion. Additionally, advanced age reduces tissue healing and increases susceptibility to infections, elevating the likelihood of anastomotic leaks [13].

Surgical Factors

Several surgical factors influence the development of anastomotic leaks. The type of anastomosis (cervical, intrathoracic, or intra-abdominal) can affect the risk, with cervical anastomosis generally considered a higher risk due to limited blood supply and potential tension [14]. The surgical approach, whether open or minimally invasive, has considerations, but studies often do not show a significant difference in leakage incidence. Omentoplasty, the placement of omental tissue, helps prevent leaks by forming adhesions and localizing potential inflammations. End-to-end anastomosis is associated with lower leakage incidence than end-to-side, especially in cervical anastomosis [15]. Linear-stapled and hand-sewn techniques do not consistently show significant differences in leakage rates or postoperative outcomes. Additionally, tension on the anastomosis and compromised blood supply can impair wound healing and increase leak risk, making adequate tissue perfusion and tension-free anastomosis crucial [16].

Perioperative Care Factors

Perioperative care factors significantly impact the development of anastomotic leaks. Adequate preoperative patient optimization, including nutritional support and comorbidity management, can mitigate the risk. The impact of neoadjuvant chemoradiation on leaks varies, with conflicting evidence regarding safe radiation doses. In cervical anastomosis, an average radiation dose of 24.2 Gy, 41.1 Gy, and 45 Gy showed no significant association with anastomotic leaks [4,17,18].

Balancing intraoperative fluid administration is crucial, with goal-directed therapy proposed to optimize fluid management. Intraoperative factors such as surgical techniques, tissue handling, and hemostasis play essential roles in preventing leaks, with cervical anastomosis carrying higher risks due to factors like longer gastric conduits, compromised vascularity, and tension [7]. Surgical techniques, including end-to-end anastomosis and stapled vs. hand-sewn methods, have varying impacts on leakage rates [8]. Thoracic epidural analgesia (TEA) can positively affect intestinal perfusion but should be carefully administered to avoid excessive sympathetic block. Ephedrine administration may help improve tissue perfusion and reduce ischemic conditions at the anastomotic site [9]. Vigilant postoperative care, including drainage, wound care, and monitoring, is crucial for early leak detection. However, cervical locations may introduce additional complications like recurrent laryngeal nerve paresis, wound infections, and extended hospital stays [19].

Clinical presentation and diagnosis

Clinical Presentation

Timely and precise diagnosis of anastomotic leaks post esophagectomy is crucial to initiate appropriate management strategies and prevent further complications [6]. The clinical presentation of these leaks can vary, encompassing signs, symptoms, and diagnostic modalities for identification. Anastomotic leaks can manifest with various signs and symptoms, dependent on their severity and location [9]. Common clinical indications include fever and sepsis, respiratory distress, chest pain, subcutaneous emphysema, increased drainage output, swallowing issues, and gastrointestinal symptoms such as nausea, vomiting, and abdominal pain [12].

Diagnostic Modalities

Diagnostic modalities for identifying anastomotic leaks encompass clinical evaluation, radiological imaging (e.g., chest X-rays, contrast-enhanced CT scans, and fluoroscopy), endoscopy (utilizing esophagogastroduodenoscopy for direct visualization and contrast leakage confirmation), laboratory tests (including C-reactive protein and white blood cell count), drain fluid analysis (indicative of infection or leakage), contrast swallow studies (revealing leaks via contrast extravasation), and nuclear medicine scans (like technetium-99m scintigraphy pinpointing areas of elevated inflammation and infection) [13-16].

Multimodal Approach

A multimodal diagnostic approach combining clinical assessment, radiological imaging, and endoscopic evaluation is key to accurately diagnosing anastomotic leaks [8]. Each modality provides unique insights into the leak's severity, location, and potential complications. Timeliness in diagnosis is critical for better patient outcomes [9]. In summary, identifying anastomotic leaks after esophagectomy demands clinical vigilance and a multimodal approach, integrating clinical evaluation, imaging, endoscopy, and laboratory tests to confirm their presence and severity, thereby guiding effective management and enhancing patient outcomes [20,21].

Management

Conservative Management

Conservative management is vital in addressing anastomotic leaks post esophagectomy, especially when surgery poses substantial risks or for less severe leaks [19]. This approach aims to foster natural healing, infection control, and support through a combination of measures, including nutritional support, antibiotics, drainage, and endoscopic therapies [8]. In recent years, non-operative management has gained prominence, demonstrating comparable outcomes to surgical interventions regarding leak closure time [7]. Nutritional support is essential, with enteral nutrition preferred for gut maintenance. Antibiotics are employed to prevent and control infections, adapted based on local resistance patterns and culture results. Drainage of fluid collections is critical, typically using surgical drains, and external drains may be used [9]. Patient education and shared decision-making enhance the success of conservative management by ensuring patients understand the strategy and the importance of follow-up care.

Surgical Management

Surgical intervention is a primary approach for severe anastomotic leaks following esophagectomy, particularly in cases of early leaks (<72 hours post-resection), failed conservative or endoscopic treatment, and unstable patients [22]. Surgical options aim to eliminate the leak source, control infection, and restore anastomosis integrity, especially for cervical and intrathoracic anastomosis with non-contained mediastinitis, empyema, systemic sepsis, or gastric conduit necrosis. Indications include severe leaks, hemodynamic instability due to sepsis, failure of conservative management, and the presence of abscesses or necrotic tissue [22,23].

Surgical Techniques

Early leaks without thoracic empyema can be managed thoracoscopically with debridement and mediastinal drainage [8]. Cases with empyema or sepsis require open surgery involving lung decortication, leakage drainage, and assessment of gastric tube integrity. Surgical management considers conduit ischemia and necrosis [16]. Preservation and suturing of the gastric tube are possible for non-necrotic disruptions. Necrosis of the gastric fundus tip may necessitate tissue resection and immediate reanastomosis [4-8]. Severe sepsis due to diffuse ischemia or necrosis may require a rethoracotomy or cervicotomy, anastomosis takedown, and gastric tube resection. Extensive necrosis may mandate a temporary cervical esophagostomy and later gastrointestinal continuity restoration using colon or jejunum interposition [9].

Primary resection and re-anastomosis: Suitable for localized leaks with healthy surrounding tissues, this technique involves removing the leak-containing esophageal section and creating a fresh anastomosis with the remaining healthy portions [24].

Diversion procedures: Used in complex or severe leaks, these procedures redirect ingested material flow away from the leak, promoting healing. Options include cervical esophagostomy, gastrostomy, or jejunostomy to establish alternate nutrition routes.

Stent placement: It involves inserting a stent at the leak site, providing support for tissue healing [25,26]. This method is ideal for small, localized leaks or high-risk surgical scenarios.

Omental or muscle flap coverage: These approaches are employed when tissue loss or compromised tissue quality is present. Omental or muscle flaps provide well-vascularized tissue to cover the leak site, facilitating healing and reducing the risk of further leakage.

Thoracotomy or laparotomy: Open surgical approaches like thoracotomy or laparotomy are used for complex leaks, extensive tissue loss, or situations where minimally invasive methods are unsuitable. These approaches allow for thorough repair and management of complications [27].

Minimally Invasive Techniques

Video-assisted thoracoscopic surgery (VATS): VATS is a minimally invasive technique used to access the thoracic cavity. Small incisions are made in the chest, and a thoracoscope (a camera) and specialized instruments are inserted through these incisions. VATS provides visual access to the thoracic area, allowing the surgeon to repair the leak with minimal tissue disruption. VATS offers advantages like reduced postoperative pain, shorter hospital stays, and faster recovery compared to open surgery [28].

Laparoscopic Approaches

Laparoscopic techniques involve accessing the abdominal cavity through small incisions. Instruments and a laparoscope are inserted to visualize and repair the leak. Laparoscopic approaches are suitable for leaks located in the abdominal portion of the esophagus. Like VATS, laparoscopic procedures offer benefits such as decreased postoperative pain and quicker recovery [29].

Endoscopic Approaches

Stent-over-sponge technique: The stent-over-sponge (SOS) technique is an innovative approach for managing anastomotic leaks after esophagectomy. It combines stent placement with absorbent sponges to promote healing and prevent leakage. SOS is suitable for uncontained leaks, particularly when endosponge therapy fails, and it offers a dual-action approach, providing both mechanical support and wound healing benefits. Recent studies suggest its potential as a first-line treatment for complex leaks [30]. The procedure involves evaluating the patient's suitability, endoscopic placement of an antimicrobial-impregnated sponge directly over the leak, followed by stent placement, which expands to create a stable scaffold holding the sponge in place. Advantages include its dual approach, immediate closure, wound healing support, and minimally invasive nature. Considerations involve the need for expertise, patient suitability, material choices, stent selection, regular follow-up, and potential complications like sponge migration, stent-related issues, and infection [31].

Sealants in Anastomotic Leak Management

Sealants, such as fibrin glue and cyanoacrylate sealants, are used to seal small tissue defects like anastomotic leaks after esophagectomy. These products create a barrier to prevent fluid and air leakage, facilitating healing while reducing infection risk. Sealants are suitable for small leakages (<15 mm) or residual fistulas after endoluminal vacuum-assisted closure. Limited studies are available, and direct comparisons between sealant types are lacking [32]. Fibrin glue and cyanoacrylate sealants have been effective, even in cervical areas [33]. Combining sealants with other techniques, like clips or Vicryl plugs, has shown promise even for leakages > 15 mm [34]. Sealants come in various types, including fibrin, synthetic, hydrogels, and cyanoacrylate. Advantages include minimally invasive application, immediate barrier formation, versatility, and reduced infection risk [33]. Considerations involve the need for expertise, patient suitability, sealant properties matching leak characteristics, regular follow-up, and potential complications like allergies or tissue irritation [32].

Stent Placement

Stent placement is a minimally invasive method for managing anastomotic leaks after esophagectomy. Stents, typically made of self-expandable metallic or covered materials, provide mechanical support to the compromised tissue, promoting healing while preventing fluid and air leakage [35]. Fully covered self-expanding metal stents (FSEMS) are preferred due to lower complications. The median stenting time for healing is four to eight weeks, with better results in smaller leaks diagnosed sooner. Stents are suitable for leaks extending up to 70% of the circumference but are now discouraged for circumferences over 30% [36]. Stents are typically left in place for two to four weeks and then removed, with an endoscopic evaluation of the leak area for closure [35,36]. Stents have similar clinical success rates (self-expanding plastic stents (84%), FSEMS (85%), and partially covered self-expanding metal stents (86%)), although plastic stents require longer placement and have a higher migration rate (ranging from 16% to 62%) [37,38]. Challenges include stent migration, complications during placement/removal, appropriate case selection, stent removal, complexity of extensive leaks, and specialized stent limitations, particularly in the upper esophagus. Stent failure risks involve leak length > 6 cm, late stent positioning (more than two days after leak development), and cervical location [38].

Endoscopic Clips (Over-the-Scope Clips)

Endoscopic clips, specifically over-the-scope clips (OTSC), offer a minimally invasive and versatile approach for managing anastomotic leaks after esophagectomy. OTSCs, designed for efficient tissue closure, are suitable for small, acute leaks with minimal inflammation and perforations up to 15 mm, although success rates decrease for lesions > 13 mm [39]. They provide full-thickness wall closure with a wider mouth, larger clip area, and higher compressive force due to their design. Studies demonstrate successful GI anastomotic leak closure in 73.3% of patients, with higher success rates for primary placement [40]. The study on esophageal perforation closure with endoluminal clips found a clinical success rate of 56-100%. OTSCs are minimally invasive, ensuring immediate, secure, and strong tissue closure while causing minimal disruption to surrounding tissues. They are valuable for complex leak cases [41]. However, successful outcomes rely on expertise in endoscopy and OTSC deployment. Patient selection based on leak characteristics and location is crucial. Rare complications like clip migration, tissue injury, bleeding, and inflammation may occur. Complex cases may require alternative approaches or multiple clips, and regular follow-up is essential for monitoring OTSC stability and detecting issues [40,41].

Endoscopic Vacuum-Assisted Closure

Endoscopic vacuum-assisted closure (EVAC) is a minimally invasive method that uses negative pressure (about -100 to -125 mmHg) through endoscopy to manage anastomotic leaks post esophagectomy [42]. It promotes healing, removes exudate, and closes leaks, offering an alternative to surgery. Indications include large anastomotic breakdowns, local contamination, chronic fistulas, and abscesses. EVAC involves applying suction through a drainage tube, achieving several benefits like exudate removal, wound healing stimulation, cell migration, and tissue regeneration. Vacuum-sealed sponges are changed every few days (usually five to six times per patient), maintaining sterility and effective pressure. Monitoring via endoscopy and imaging guides adjustments to the treatment plan. EVAC concludes when stable granulation tissue covers the wound, with healing times typically spanning 12 to 36 days [43-46].

EVAC therapy has shown effectiveness in treating anastomotic leaks after upper and lower gastrointestinal surgeries, achieving a 94.2% healing rate for various indications. EVAC outperforms stent placement with an 84.4% closure rate compared to 53.8% for stents. It attains success rates between 86% and 100% for intrathoracic anastomotic leaks but has limited evidence for cervical leaks [44,45,47]. EVAC offers advantages such as faster clinical success, shorter hospital stays, and fewer complications than stent placement, although both modalities share similar mortality rates, EVAC has a shorter treatment duration [41,45].

Considerations and limitations of EVAC encompass the necessity for a skilled healthcare team with expertise in endoscopy and negative pressure systems, meticulous patient selection based on leak characteristics and patient factors, and the importance of regular monitoring through endoscopic evaluations and imaging studies [46,47]. Potential complications include bleeding, infection, and discomfort. While EVT is effective in closing anastomotic leaks, it involves frequent sponge replacement, which can be burdensome for patients and healthcare systems. Prolonged use may lead to complications such as sponge dislocation and erosion into adjacent vital structures. Stricture formation, necessitating dilation, is a common complication due to aggressive granulation tissue growth, while rare complications (around 10%) include bleeding from sponge erosion, bronchoesophageal fistula formation, mucosal tear during sponge removal, sponge dislocation, and detachment [44,48,49].

A modified EVAC technique has been introduced, where the nasogastric tube is passed into the esophagus through an existing intrapleural drain tract, allowing easier sponge placement, exchange, and oral intake during treatment [49].

*Endoscopic Suturing (OverStitch* *)*

Endoscopic suturing (OverStitch; Apollo Endosurgery, Austin, TX) is a technique employed to manage anastomotic leaks post esophagectomy. It allows precise suture placement within the gastrointestinal tract, facilitating endoscopic-guided closure of tissue defects and leaks. The OverStitch system, mounted on a double-channel endoscope, permits full-thickness suture placement for gastrointestinal defect closure [50]. While OverStitch has been used for leak closure in other procedures, its application to esophagectomy-related leaks is limited. A study combined endoscopic suturing with partially covered self-expanding metal stents (pcSEMS) to address esophagectomy-related anastomotic leaks, but long-term evidence is lacking [50]. Suturing techniques for anastomotic leak closure lack established effectiveness and substantial investigation. OverStitch might be suitable for leaks in the middle or distal esophagus, yet its application for anastomotic leaks remains limited and anecdotal [51].

Its advantages lie in the precision it offers, allowing for accurate placement of sutures within the gastrointestinal tract, thus ensuring the precise closure of tissue defects and leaks under endoscopic guidance. This immediate closure reduces the risk of further complications and promotes the healing process [51]. The method is highly customizable, permitting the placement of multiple sutures tailored to the size and shape of the tissue defect. Importantly, endoscopic suturing is a minimally invasive approach, avoiding the need for more extensive surgical procedures [45]. However, successful outcomes rely on the expertise of skilled endoscopists experienced in suturing techniques. Patient selection is crucial, taking into account factors such as leak characteristics and location. The choice of suture materials should align with tissue characteristics and surgeon preference, and precise knot tying is paramount to ensure effective leak closure while avoiding issues like suture loosening and leakage. Regular follow-up is necessary to monitor suture stability and healing progress, and potential complications, including suture breakage, tissue injury, and inadequate closure, must be considered and managed appropriately [45,52].

Hybrid Approaches

Hybrid approaches combine VATS and laparoscopic techniques to address leaks that extend across both the thoracic and abdominal regions. These approaches provide comprehensive access to the entire esophagus while still benefiting from minimally invasive techniques [53].

Novel and Emerging Techniques

Emerging techniques in the management of anastomotic leaks post esophagectomy include a novel method employing a large-bore 36 Fr reversed nasogastric tube retrogradely placed via a gastrotomy about 5 centimeters proximal to the leak, which has been utilized for leaks ranging from 10% to 50% of anastomotic circumference [54]. Ischemic preconditioning of the stomach, aimed at enhancing gastric perfusion before resection, has been investigated. This approach entails the ligation or occlusion of the left gastric artery, typically performed at least two weeks before resection using laparoscopic arterial ligation or radiological arterial embolization. While outcomes have shown variability, a recent meta-analysis did not find sufficient evidence for reduced anastomotic leak rates. Nevertheless, it affirmed the feasibility of these interventions and called for randomized trials [29,55,56]. Tissue engineering offers potential by combining cells, biomaterials, and growth factors to create biological grafts that can bridge and reinforce defects, promoting healing. One example is the use of a thick AlloDerm patch, demonstrating success in salvaging dehisced conduits when sutured into the affected area along with concomitant stenting [57]. Biodegradable stents made of polydioxanone monofilament, which degrade through hydrolysis, have shown promise in closing anastomotic leaks with success rates of 80-85%, although they come with higher costs and potential side effects [58,59]. A unique "chimera" stent, combining an 18-mm diameter fcSEMS with a 28-34-mm diameter fully covered self-expandable colonic metal stent, was effective in preventing gastric reflux and was removed after three weeks of placement [60].

Prevention strategies

Preventing anastomotic leaks after esophagectomy involves comprehensive strategies spanning patient selection, surgical techniques, and perioperative care [23]. Key preventive measures include optimizing patient health by managing comorbidities, promoting smoking cessation, and ensuring adequate nutrition. Surgeon experience is crucial, as is preserving blood supply, creating tension-free anastomosis, and conducting intraoperative leak testing [31]. Enhanced Recovery After Surgery (ERAS) protocols, a multidisciplinary approach, early mobilization, optimized pain management, and strict infection control all play vital roles [37]. Vigilant monitoring, early detection through clinical assessment and imaging studies, minimizing tissue ischemia, intraoperative imaging like fluorescence imaging, exploring innovative suturing techniques and bioresorbable sutures, postoperative surveillance via regular endoscopy, and implementing quality improvement initiatives including audits and learning from complications are key elements in effective prevention strategies [40,45].

Discussion

Anastomotic leaks following esophagectomy pose substantial clinical and financial burdens, impacting patient well-being, mortality, and healthcare costs [9,21,22]. Surgeons continually refine techniques to prevent and manage these leaks, emphasizing the critical importance of early diagnosis [29,30]. While routine imaging for asymptomatic patients may have limited value and resource constraints, emerging treatment modalities demand surgical skill and a comprehensive understanding [4,15]. Individualized strategies guide current clinical management, highlighting the need for international, evidence-based guidelines. The Esophagectomy Complications Consensus Group (ECCG) system provides outcome benchmarks but could benefit from standardized leak descriptions [61]. Real-time perfusion monitoring techniques, like indocyanine green fluorescence angiography, show promise [62]. Limitations in the literature involve the scarcity of specific data on anastomotic leak management alone, typically encompassing various interventions for esophagectomy patients with varying conditions [35,39,51]. Nevertheless, this review focuses on anastomotic leaks. Urgent research is needed, particularly for refining risk factors and developing individual risk stratification tools [9]. Management trends lean toward conservative and endoscopic approaches for anastomotic leaks, reserving surgery for severe cases, and weighing the benefits of continued enteral nutrition through endoluminal stenting against potential complications, like stent migration [8,9,10]. Promising results emerge from endoluminal vacuum therapy (EVAC) in closing anastomotic leaks, though further research, especially for complex cases, is warranted [42]. In a continuously evolving medical landscape, innovative methods are likely to emerge to address anastomotic leaks with the aim of complete elimination.

## Conclusions

In conclusion, managing esophageal anastomotic leaks post surgery is a multifaceted challenge with diverse treatment options. Endoscopic interventions offer promise with their minimally invasive nature, reduced morbidity, and shorter hospital stays, while surgical approaches remain essential for complex cases. Tailoring treatment to individual patient factors, multidisciplinary collaboration, and prevention strategies are crucial. Ongoing research is necessary to refine guidelines, emphasizing the need for standardized protocols. Ultimately, the optimal approach should prioritize patient characteristics, considering the benefits and risks of each intervention, aiming to enhance patient care in this complex clinical scenario.
